# Perspective on clinical high-risk for psychosis in Africa

**DOI:** 10.3389/fpsyt.2023.1226012

**Published:** 2023-09-08

**Authors:** Sewanu Awhangansi, Adeniran Okewole, Philip John Archard, Michelle O’Reilly

**Affiliations:** ^1^Leicestershire Partnership NHS Trust, Leicester, United Kingdom; ^2^Neuropsychiatric Hospital Aro, Abeokuta, Nigeria; ^3^Pembroke College, University of Cambridge, Cambridge, United Kingdom; ^4^University of Leicester, Leicester, United Kingdom; ^5^Tavistock and Portman NHS Foundation Trust, London, United Kingdom

**Keywords:** Africa, attenuated psychosis syndrome, clinical high risk for psychosis, early onset psychosis, psychotic-like experiences

## Abstract

Clinical High Risk for Psychosis has evolved in recent years as a conceptual and clinical entity, representing a shift in focus from the syndromal psychosis state to a recognition of the pre-psychotic state as a period of potential preventive intervention. Much existing evidence has been generated from well-resourced countries, with a more limited body of literature available from Africa and other Majority World countries. Against a backdrop of prevailing systemic challenges, it is necessary to appraise the state of knowledge on Clinical High Risk for Psychosis in Africa. In this perspective article, we cover epidemiology, risk factors, predictors of psychosis conversion, as well as an overview of sociocultural factors, notably stigma, and the barriers to mental health services in African settings. We discuss existing and promising assessment approaches and reflect on preventive and early intervention strategies. We conclude with recommendations including the need for more clinical, longitudinal, and collaborative research anchored in an integrative transdisciplinary approach. We highlight the need for more culturally valid assessment tools and strategies to improve access to and utilization of services while also reducing stigma.

## Introduction

Psychosis is an umbrella term for a group of conditions (including schizophrenia and mood disorders with psychosis) characterized by delusions, hallucinations and disorganized thought, speech, and behavior. Psychotic conditions are associated with a wide range of adverse psychosocial outcomes (1) and contribute in significant ways to the global burden of disease (2). Individuals with psychotic illness tend to have a lower quality of life (3, 4) and shorter life expectancy—approximately 10–15 years less than the non-afflicted population (5, 6). Due to the chronicity, debilitating nature and overall poor prognostic outlook of psychotic conditions, better understanding of the progression of the disorder from a clinical high-risk of psychosis (CHR-P) to meeting full criteria for psychosis is an important area of empirical concern (7).

The concept of CHR-P has evolved over time, with a variety of associated terms used to denote this stage of the condition of psychosis, including at-risk state, attenuated psychosis syndrome (APS), psychotic like experiences (PLE) and prodromal symptoms of psychosis. Yet, regardless of terminology, CHR-P research is not well developed in Africa. CHR-P has been described as a syndrome with imminent risk of transition to overt psychotic states characterized by subclinical symptoms, including gradual cognitive decline (8, 9) and functional impairment (10). According to Fusar-Poli et al. (11), the concept captures, to a large extent, a “pre-psychotic” state whereby prodromal symptoms are present alongside risk factors for psychosis (12, 13).

While most of the evidence regarding the etiology, presentation, course (pre-psychotic to chronic psychotic states) and outcome of psychosis is largely drawn from well-resourced countries, there is growing awareness of the need to bridge the knowledge gap and expand understanding of psychosis in Africa, as well as Majority World countries elsewhere. Crucially, exploring how psychosis develops across different socio-cultural contexts increases the diversity of global data available, ensuring that African science and people are equitably represented in any advances in psychosis research (14). Moreover, such exploration affords a more contextualized understanding of etiological processes, early identification, prognostication, and tailored intervention based on individual risk profiles in Africa (15).

Within a general climate of poor health financing and infrastructure, mental health services are underfunded, understaffed and underprioritized across most of Africa (16, 17). There is therefore significant clinical and economic utility in the early detection and provision of targeted intervention strategies to prevent or delay psychosis onset in the at-risk group (10, 18).

In this article, we offer a perspective review of current knowledge, addressing the role of different factors and issues, including culture, stigma, and economic deprivation, as well as barriers to accessing mental health services in Africa for those at high-risk for psychosis. Importantly also, we reflect on the gaps in what is currently known in the field and outline ways in which future researchers, clinicians, and policy makers in Africa might take steps to progress research, practice, and policy.

## Epidemiology

Globally, the CHR-P state has steadily gained recognition—it is now a diagnostic category in the fifth edition of the Diagnostic and Statistical Manual of Mental Disorders (DSM-5) (19) while also proving prognostically useful (10, 20). Yet, in Africa, there is still a dearth of literature, with the vast majority of the available knowledge in the field coming from research conducted in Kenya (15, 21–27). Findings from the small body of available studies in Africa indicate that the presence of psychosis-risk symptoms is relatively common in the population, with high rates of psychotic-like experiences ranging from 1.8 to 45.5%, reported among Kenyan children, adolescents, and young adults, using various psychosis-risk screening tools (21, 22, 24). A high prevalence rate of 20.9% for prodromal psychotic symptoms was reported among secondary school students in Nigeria using the Brief version of the Prodromal Questionnaire (PQ-B) (28). In another study, involving a population of Nigerian school-going adolescents, a 10.5% prevalence of clinically significant psychotic-like experience (PLE) symptoms was reported using the 16-item version of the Prodromal Questionnaire (PQ-16) (29).

These studies were conducted among non-help seeking younger age group samples in school and community settings as there is robust evidence that psychosis onset is typically during adolescence or early adulthood (30, 31). The wide variation in prevalence rates is also consistent with findings from a cross-national global study (32), but the use of different assessment tools, most of which were originally developed in and for developed countries, might limit cross-cultural application. As Mamah et al. (15) suggest, local development of more nuanced, culturally sensitive assessment tools should afford a more consistent conceptualization of the CHR-P state in African settings.

Nonetheless, these prevalence figures appear to be generally higher than the 1.7% prevalence rate reported among non-African general population samples in one systematic review and meta-analytic study (33), with the possibility of an overestimation using the locally adapted assessment tools originally intended for Western populations (15). Indeed, strikingly, a comparatively lower prevalence rate was reported among Rwandan students, with about 5.35% of older Rwandan students (born pre-conflict, that is, before the Rwandan genocide of 1994) meeting the threshold for clinical high-risk of psychosis using the Washington Early Recognition Center for Affectivity and Psychosis (WERCAP) Screen (a CHR-P screening tool) compared with 3.19% of younger Rwandans (born post-conflict, after 1994) (27). One would expect higher rates of psychosis-risk in such a population given the documented links between experience of armed conflict, trauma exposure and subsequent development of psychotic disorder (34, 35). Plausible explanations for the reduced prevalence rates in psychosis risk among younger Rwandan students born after the war might include the wide scale implementation of mental health reform and policy in the years since the genocide in Rwanda (36) and development of effective psychosocial intervention programs, including community-based workshops (37). While the older cohort had an average age of 2 years when the war ended, they lived through the same post-war environment as their younger peers, but studies so far have only been able to link psychosis-risk to time of birth during the war, rather than a simple effect of age (27), and longitudinal studies are needed to clarify causal links.

The absence of longitudinal studies means that it is difficult to draw robust conclusions regarding conversion rates of the psychosis high-risk states in Africa. The only longitudinal study that has been carried out to date reported a 3.8% conversion rate after a 20-month follow up (15) which is much lower than the mean transition risk of 36% after 3 years, independent of the psychometric instrument used, reported in a meta-analytic study by Fusar-Poli et al. (38). The wide gap in transition rate is understandable because participants in the longitudinal study by Mamah et al. (15) comprised community samples of adolescent secondary school students whose profile was compared with the samples of help-seeking individuals in clinical settings in the meta-analysis. More longitudinal studies are needed in Africa to effectively identify this high-risk group and determine socio-demographic and clinical factors associated with transition likelihood. Such longitudinal studies will also potentially afford useful insights into the clinical and functional characteristics of the non-converting group.

## Risk factors

Several risk factors for CHR-P have been reported. For instance, childhood adversity has been strongly linked with multiple mental health problems, including the gradual development of at-risk psychotic state (39, 40). This link may be due to the neurodevelopmental effects of trauma during childhood and damage to the stress regulation mechanisms in the hypothalamic pituitary adrenal (HPA) axis, as well as in attachment security and capacity for mentalization (41, 42), with high stress sensitivity found in those with CHR-P (43–45). The findings of studies of CHR-P in Africa have reflected what is known about ways early childhood trauma and adverse psychosocial stressors could be strong predisposing factors to the development of psychosis (28). Future research in Africa exploring the link between CHR-P and biomarkers of stress sensitivity like cortisol levels as well as other behavioral markers may prove useful in illness prediction for early intervention and determining effectiveness of such interventions (15).

Another factor is stress sensitivity which is especially important in adolescence due to the specific stressors experienced by this age group. For example, there are various school-related stressors, familial challenges, identity development, and peer relations to manage. One growing concern in relation to adolescent stress across the globe is that of bullying (46), an experience widely acknowledged to impact adolescent mental health (47). This is also recognized in the African context, and in relation to CHR-P. For example, the experience of bullying victimization was found among Nigerian adolescent students to be associated with CHR-P (28). The direction of causality (or nature of the bidirectional influence) is, however, yet to be determined due to the limited African literature. On the one hand, bullying victimization by peers is an established risk factor in the development of psychosis in a dose–response relationship (48). On the other, some children who present with early CHR-P symptoms have personal developmental characteristics (like delayed motor milestones, poor expressive language ability, social maladjustment, neuropsychological deficits, and intellectual impairments) that make them more vulnerable to victimization (49).

Psychiatric comorbidity is a risk factor that was observed commonly in the few existing studies in Africa. In their longitudinal study among Kenyan adolescents, Mamah et al. (15) reported an increased propensity for the presence of other psychiatric co-morbidities among the high-risk for psychosis group. Externalizing disorders in the form of conduct disorder and oppositional defiant disorder were the most prevalent conditions in their study. Adewuya et al. (29) found an independent association between existing diagnosis of depression and CHR-P among adolescents in Nigeria. Similarly, Okewole et al. (28) found that CHR-P symptoms were predicted by emotional and psychological distress, with higher levels of internalizing and externalizing symptom scores noted in their study sample. This finding is not unique to Africa, as it has also been noted in other populations (50), indicating a need for comprehensive assessments to be routinely completed for young people who display internalizing and externalizing symptoms, especially in schools and primary care settings, to aid early identification and timely intervention.

Furthermore, neurocognitive deficits are risk factors that are quite common in CHR-P and are useful in predicting functional outcomes (51). In the North American Prodrome Longitudinal Study (NAPLS 2), the largest CHR-P study currently in existence, a small to large effect size in neurocognitive deficits was noted among CHR individuals in the working memory, declarative memory, and attention domains (52). To our knowledge, only two published studies (15, 53) have assessed neurocognitive functioning among CHR-P subjects in Africa. These studies reported decreased attention and increased ability for abstraction in CHR-P subjects compared to controls, a unique finding which may inform the cognitive and intellectual profiling of those with CHR-P in Africa. Cognitive deficits documented in the study were most notable when psychosis-risk status was assessed using a self-report measure rather than a structured interview-based assessment. This finding may be indicative of the significant role of internalized stigma and other socio-cultural barriers in accessing care and treatment for mental health problems in Africa (54).

Moreso, personality traits, as well as cognitive traits, are independent and informative endophenotypes that have been associated with the development of schizophrenia (55) and have been reported among first degree relatives of individuals at risk of psychosis in developed countries (56, 57). To our knowledge, the only study in an African context that investigated the relationship between personality and psychosis found distinct personality traits among young people at CHR-P (23). High novelty seeking and low reward dependence were the most notable traits observed, while a schizotypal character profile of low self-directedness, low cooperativeness and high self-transcendence were the remarkable traits identified in subjects considered to be at high risk for psychosis. Novelty seeking is strongly correlated with engagement in substance misuse, including excessive cannabis use, which is a risk factor for psychosis (58). Further examination of personality trait endophenotypes as part of genetic studies in Africa could provide novel etiological insights, while longitudinal studies will increase understanding regarding causal mechanisms.

## Predictors of psychosis conversion

The shortage of longitudinal research hinders our capacity to accurately predict psychosis conversion among CHR-P subjects in Africa and elsewhere. The only longitudinal study in Africa to date found a 3.8% rate of conversion to psychosis at 7-month follow up for those deemed to be high-risk in Kenya. There was no further conversion, with the rate maintained at the 20-month follow up. None of the low-risk participants converted to psychosis throughout the study. Despite the much lower rate of conversion compared with reported rates of 16–54% after 1–2.5 years in Western countries (59, 60) and a 17% conversion rate at 3-month follow-up in a Tunisian study (61), the findings are quite instructive for our current understanding as well as for future researchers. Some of the explanations provided for the wide variance include the younger mean age of study participants in the African study compared with the Western studies (62), which suggest the possibility that psychosis conversion occurred outside the study timeframe. What is more, it has been established that the risk of developing psychosis in high-risk patients increases as follow-up time increases (38), with the risk of transition thought to be most pronounced at 24-months following initial presentation (59).

Interestingly, severity of disorganized communication at baseline was the only predictor of psychosis conversion found in the Kenyan study. This is consistent with existing knowledge about predictors of psychosis onset outside of Africa (63, 64). Disorganized speech is a clinical indicator that can suggest underlying formal thought disorder, a cardinal sign of psychosis. The presence of disorganized communication through the prodromal phase and its persistence after psychosis onset, together with findings that disorganized communication aggregates in families of people with psychosis therefore makes it a potential candidate as a behavioral endophenotype for schizophrenia risk (65). Other traits that have an established link with psychosis conversion include paranoia, low social functioning, substance misuse and familial risk (62, 66).

Notably, a significantly lower risk of transition to psychosis was observed among high-risk groups receiving active intervention (pharmacological, psychological, nutritional, and complex psychosocial interventions) compared with those that were not (38, 67). Although evidence of sustained long-term benefits for any specific intervention is not conclusive (68), this evidence supports a need to expand existing mental health services in Africa to include early intervention provision. While research focus in Africa has largely been about understanding risk factors for CHR-P, there has been minimal emphasis on identifying protective factors. Most of what we know about protective factors for CHR-P are from outside Africa and are largely grouped as individual, family, and social factors (69, 70). Some of the individual factors are having a relatively higher intelligence quotient (71), being resilient and having personality traits of extraversion, openness, agreeableness, and conscientiousness (72, 73). Family factors include a positive family environment where there are positive remarks and warmth from caregivers (74), proper family planning with maximum of four children and 2 years between them, consistent parenting style with clear boundaries and a healthy relationship with siblings (75, 76). Living in a cohesive community with adequate social support have also been found to be protective (71). Though these factors on the surface appear translatable to African settings, there is need for empirical research that can put them in context. Future research in Africa will therefore need to be larger in scope and scale, that is, longitudinal studies that extensively assess a broader complement of behavioral traits and other factors (including genetics) that are known to increase risk of psychosis transition, while also evaluating interventions and protective factors. Young people will also need to be engaged in qualitative research to better hear their voices and understand their experiences.

## Sociocultural factors, stigma and barriers in access to mental health care

Much has been written regarding the mental illness burden and treatment gap in Africa (2, 77, 78) with concerns raised regarding how little attention is paid to the myriad of implications this has for society (79, 80). The treatment gap, i.e., the proportion of people experiencing and living with mental illness who do not receive treatment, ranges from 75% in South Africa to over 90% in Ethiopia, Ghana, Nigeria, and many other African countries (81, 82). Findings from a recent systematic review identified multiple and varied factors responsible for this, including attitudinal, economic, physical, political, and infrastructural barriers that hinder access to and utilization of mental health services in Africa (83). The inequity in access to care is fueled by structural factors, such as poor resource allocation and prioritization, both rooted in inadequate research and policy capacity, and associated with shortages of mental health services and mental health professionals with adequate knowledge and skills (84).

Crucially, the longstanding shortages of well-trained mental health professionals have, over the past few years, become further compounded by the massive brain-drain by which the African continent is plagued. This workforce shortage contributes to the widening inequity that characterizes access to mental healthcare by those in need and ultimately compromises global efforts to scale up mental health services in resource-constrained settings of Africa (85). Research into the push and pull factors may help inform strategies for recruitment and retention of mental health professionals to curb this trend. This, by extension, will increase the opportunity for early detection and prompt intervention for CHR-P.

Widespread poverty in Africa is also a major factor, with a bidirectional link between poverty and mental illness burden well established (86). Low income, unemployment and other socio-economic stressors make it extremely difficult to afford out-of-pocket payment to access care and treatment. Resulting social drift is particularly relevant in those at CHR-P, as gradual loss of functioning is one of the earlier features noted in this group (10). More can be done through mental health advocacy and campaigns to make policy makers aware of these, so they can invest in policies that protects the mental health of those living in poverty.

Social stigma and poor mental health literacy are also major concerns in Africa (82). Persons struggling with mental health problems, and by extension those with or at high-risk for psychosis, can encounter discrimination due to several reasons, including due to cultural belief in the spiritual power afforded by prayers and traditional healers. Inadequate knowledge and negative perceptions about mental health issues can combine with a pervasive belief that experiencing mental health need is a sign of personal weakness (87) or transient experience that will resolve spontaneously without professional help (88). Many families of individuals with mental health problems resort to attempts to deal with these difficulties in isolation.

Beliefs about mental illness being a form of divine or spiritual punishment are still prevalent in some African communities, with the illness conceived of as the consequence of wicked acts committed by the individual sufferer or their relatives (87–89). Fear and shame associated with being known by neighbors and friends to have a psychiatric illness can result in families concealing symptoms and delay the seeking of medical care (90). It is recognized that issues of stigma can be deeply rooted. For instance, even among medical students and newly qualified doctors, high levels of stigmatizing attitudes toward those experiencing mental health problems have been reported (91, 92). While these observations may refer to mental health problems in general, they are largely applicable to the CHR-P group. There is therefore wide scope for carefully considered mental health literacy and anti-stigma campaigns as a means of helping foster community acceptance and support for those in CHR-P group.

## Assessment approaches

If early detection and intervention hold the keys to preventing or delaying transition to fully developed psychosis, it must be recognized that the tools for assessment were originally developed for Western cultures and only a few have now been validated for use in Africa. For instance, Braham et al. (93) reported good construct and concurrent validity of the Arabic version of the Comprehensive Assessment of At-Risk Mental States (CAARMS) with the Positive and Negative Syndrome Scale (PANSS) in Tunisia. Similarly, the Prodromal Questionnaire – Brief version (PQ-B) has also been validated among secondary school students in Abeokuta, Nigeria (28) showing good concurrent validity with the Structured Interview for Prodromal Syndromes (SIPS). The Washington Early Recognition Center Affectivity and Psychosis (WERCAP) Screen has been shown to be a valid screening tool for affectivity and psychosis in Kenya (25) while the modified version of the Prevention through Risk Identification, Management, and Education (mPRIME) did not show validity as an effective screener for individuals at-risk of psychosis in Kenya (26).

These efforts are commendable, but the use of different assessment tools that are not always cross-culturally applicable, may result in wide variance in reported prevalence rates (32). Thus, the development of culturally sensitive assessment tools that are better able to identify the CHR-P state across Africa is important (15).

## Prevention and early intervention

The interventions most suited to the African context are those that follow a primary prevention approach that target: the general population (i.e., universal), people at higher-than-average risk of developing mental health problems (i.e., selective), and individuals with emerging or subthreshold manifestations of mental illness (i.e., indicated) (94, 95). The conceptualization of CHR-P for an indicated preventive approach focusses on prevention of psychosis and improving outcomes. There is robust evidence that early interventions improve overall functioning, reduce symptomatology, and lower risk of transition to fully developed psychosis in those at high-risk of psychosis (96). Though still a fledgling research and clinical field globally, CHR-P services have been implemented on different continents, with Africa contributing the least (97).

A practical universal prevention strategy that can be well suited to resource-constrained settings in Africa must, by necessity, involve increasing political will and commitment by governments to address potentially modifiable social determinants of psychosis risk, like poverty and unemployment, social deprivation, widespread illiteracy, exposure to environmental and interpersonal traumatic events, and expanding social and cultural capital (98). Such a strategy will require large-scale health promotional campaigns as well as strengthening of legislation and mental health policies that will guide development of programs and services and ensure that significantly more funds, than the current average of 1%, is allocated to mental health in national health budgets (99).

In their systematic review, Estradé et al. (94) highlighted that the selective prevention approaches for those at higher-than-average risk of developing psychosis tend to target different foci or domains known to foster positive mental health: skills in self-management, family and other important relationships, social skills, occupational or academic performance, intellectual functioning, and general quality of life. Some of the positive mental health promotional interventions that have been used with healthy individuals, with a varying degree of effectiveness, include psychoeducation, family support, interpersonal psychotherapy, cognitive behavioral therapy, resilience training, animal assisted therapy, physical therapy, art therapy, among others (100). As many of these interventions can be delivered by lay persons, clinicians, researchers, and policy makers in Africa can find innovative ways to consolidate relevant evidence for widespread application.

The strategic approach for indicated prevention is for prompt detection of CHR-P individuals and the swift provision of tailored intervention based on individual needs. Some of the interventions that have been implemented globally, with varying degree of success, include close clinical monitoring, crisis management, supportive therapy, structured psychotherapy, and pharmacological treatment (97). Pharmacological treatment options that have been suggested include mood regulators, antidepressants, antipsychotics and nutritional supplements such as Omega-3 fatty acids and D-Serine (101). In their systematic review of indicated interventions for adolescents and adults with psychosis in Africa, Hunt et al. (102) reported that most of the interventions were categorized as involving some form of psychoeducation, awareness, and social support, while the second most common intervention category were those offering clinical support like medication prescription, adherence support, clinical monitoring, and appointment reminders. Psychologically based interventions followed by traditional and faith-based healing were the other categories identified. It is worth mentioning that the clinical high-risk program (CHiRP) developed in Tunisia can be a cost-effective prevention strategy that could be scaled up and replicated across Africa, while remaining integrated in existing poorly resourced health systems structure.

## Recommendations

Although still an underdeveloped field in Africa, CHR-P has been receiving increasing clinical and research interest over the past few years, but this has been accompanied by a lack of administrative and policy commitment. More research is still needed involving clinic and community samples to obtain more data on etiopathogenesis, predictive risk factors, and pattern of presentation. More longitudinal studies across the continent will help to extensively characterize identified high-risk groups and factors associated with the likelihood of transition to full psychosis. These studies will also provide valuable insights regarding the clinical and functional characteristics of the non-converting group. Research collaborations with investigators in better resourced settings can be crucial for this. Findings from empirical research can strengthen the evidence-base of need for service development.

Furthermore, implementing an integrative transdisciplinary clinical research model, that incorporates different but complementary strategies, holds a lot of promise (103). Such pluralistic strategies must address biological, psychological, and social factors to obtain a holistic view, factoring individual characteristics, alongside familial, cultural and transcultural contexts (104). Based on experience elsewhere, research incorporating qualitative methods and already available clinical data can play a valuable role in resource-constrained settings, for example, in conceptualizing stages of recovery, young people’s experiences, and the practices of mental health professionals in assessment and treatment (105–108). Such methods can help centralize the young person’s voice, which is necessary for recognizing the unique and important contribution that this population can make to strategy and policy, while integrating new modern perspectives on children and childhood and their rights to be part of the solution (109–111).

Importantly also, the success of any strategy for improving outcomes hinge largely on accurate assessment, prediction, and detection of individuals at CHR-P. The scarcity of transculturally validated tools in Africa has resulted in an overreliance on Western-derived scales which may inadvertently induce what Guessoum et al. (104) have described as “experience measure fallacy” whereby the experience of people that is measured by these tools is shaped by Western idioms used in speaking about mental distress, relegating African cultural dimensions and contexts. Recommendations on this matter include the design of more culturally appropriate tools for CHR-P and the transcultural validation and adoption of existing CHR-P assessment instruments (104).

As Western countries are maximizing scientific advancement and integrating information technology into research and practice, the use of an automated transdiagnostic individualized risk calculator that screens medical records to detect those at risk of psychosis and refer them to standardized CHR-P assessment are currently being developed (103). African countries might take advantage of such initiatives to develop similar risk estimation tools for those at CHR-P.

Improving access to and utilization of health services by the CHR-P group are intricately linked to overall mental health services development in Africa. As mentioned, inadequate funding and poor resource allocation are major barriers to effective integration of mental health care in Africa. Implementing the World Health Organization’s recommendation for a minimum level of national funding for mental health is a good place to start (112). Multi-sectoral partnerships, between public and private not-for-profit organizations that have been successfully used in some African settings to address funding barriers and scale-up mental health services delivery can be expanded, for instance through mental health training and upskilling programs for community health workers (113).

To address issues of stigma, discrimination, misconceptions, mental health illiteracy among the public and low prioritization of mental health in the five Anglophone countries of West Africa, a Mental Health Leadership and Advocacy Program (mhLAP) was developed in Nigeria with international partners. The program aimed to build capacity for mental health leadership and advocacy while also developing stakeholder groups (specifically, service users, caregivers, non-governmental organizations, media practitioners, mental health professionals and policy makers from the different participating countries) with the ability to identify and pursue country-specific mental health service development needs and targets (114). The success of the program suggests that it may be successfully embedded and replicated in other regions of Africa.

## Data availability statement

The original contributions presented in the study are included in the article/supplementary material, further inquiries can be directed to the corresponding author.

## Author contributions

All authors listed have made a substantial, direct, and intellectual contribution to the work. SA and AO wrote the original draft of the manuscript. PA and MO’R contributed to the critical revision of the work for important intellectual content. All the authors reviewed and edited the manuscript, and all approved the final version.

## Conflict of interest

The authors declare that the research was conducted in the absence of any commercial or financial relationships that could be construed as a potential conflict of interest.

## Publisher’s note

All claims expressed in this article are solely those of the authors and do not necessarily represent those of their affiliated organizations, or those of the publisher, the editors and the reviewers. Any product that may be evaluated in this article, or claim that may be made by its manufacturer, is not guaranteed or endorsed by the publisher.
